# KDM2B, an H3K36-specific demethylase, regulates apoptotic response of GBM cells to TRAIL

**DOI:** 10.1038/cddis.2017.288

**Published:** 2017-06-29

**Authors:** Ibrahim Cagri Kurt, Ilknur Sur, Ezgi Kaya, Ahmet Cingoz, Selena Kazancioglu, Zeynep Kahya, Omer Duhan Toparlak, Filiz Senbabaoglu, Zeynep Kaya, Ezgi Ozyerli, Sercin Karahüseyinoglu, Nathan A Lack, Zeynep H Gümüs, Tamer T Onder, Tugba Bagci-Onder

**Affiliations:** 1 Brain Cancer Research and Therapy Laboratory, Koç University School of Medicine, Istanbul 34450, Turkey; 2 Koç University School of Medicine, Istanbul 34450, Turkey; 3 Vancouver Prostate Centre, University of British Columbia, Vancouver, BC, Canada; 4 Department of Genetics and Genomic Sciences, Icahn School of Medicine at Mount Sinai, New York, NY, USA; 5 Icahn Institute for Genomics and Multiscale Biology, Icahn School of Medicine at Mount Sinai, New York, NY, USA

## Abstract

Tumor necrosis factor-related apoptosis-inducing ligand (TRAIL) can selectively kill tumor cells. TRAIL resistance in cancers is associated with aberrant expression of the key components of the apoptotic program. However, how these components are regulated at the epigenetic level is not understood. In this study, we investigated novel epigenetic mechanisms regulating TRAIL response in glioblastoma multiforme (GBM) cells by a short-hairpin RNA loss-of-function screen. We interrogated 48 genes in DNA and histone modification pathways and identified KDM2B, an H3K36-specific demethylase, as a novel regulator of TRAIL response. Accordingly, silencing of KDM2B significantly enhanced TRAIL sensitivity, the activation of caspase-8, -3 and -7 and PARP cleavage. KDM2B knockdown also accelerated the apoptosis, as revealed by live-cell imaging experiments. To decipher the downstream molecular pathways regulated by KDM2B, levels of apoptosis-related genes were examined by RNA-sequencing upon KDM2B loss, which revealed derepression of proapoptotic genes Harakiri (*HRK*), *caspase-7* and death receptor 4 (*DR4*) and repression of antiapoptotic genes. The apoptosis phenotype was partly dependent on HRK upregulation, as HRK knockdown significantly abrogated the sensitization. KDM2B-silenced tumors exhibited slower growth *in vivo.* Taken together, our findings suggest a novel mechanism, where the key apoptosis components are under epigenetic control of KDM2B in GBM cells.

Glioblastoma multiforme (GBM) is the most common and aggressive primary brain tumor with a median survival of 12 months. Conventional therapeutic strategies of radiotherapy and chemotherapy are inadequate to eradicate GBMs because of the diffuse nature of GBM, acquired or innate resistance to therapy and the presence of blood–brain–barrier (BBB).^1, 2^ To alter the *status quo* that has remained unchanged over 25 years, novel targets and therapeutic strategies need to be developed.

Evasion of apoptosis is a key hallmark of cancers that exhibit resistance against therapeutics,^3^ making the reactivation of dormant apoptotic programs a favorable approach in treatment. Activating extrinsic apoptosis using death ligands, such as tumor necrosis factor-related apoptosis-inducing ligand (TRAIL), is a promising strategy because of its tumor specificity.^4^ Several TRAIL-based therapies such as recombinant human TRAIL and death receptor agonists have been developed and have shown success in preclinical models.^5^ Similarly, activating intrinsic apoptosis by inhibiting the antiapoptotic Bcl-2 proteins, Bcl-2 and Bcl-XL, with BH3 peptides has shown success in preclinical tumor models.^6, 7^ However, the major obstacle in proapoptotic therapies is innate or acquired resistance of tumor cells to the proapoptotic agents.^8^

Aberrant regulation of the apoptosis pathway components could be responsible for the failure of desired response to the proapoptotic agents.^3^ One well-characterized misregulation is the epigenetic silencing of proapoptotic genes, such as death receptor 4 (*DR4*).^9^ However, epigenetic regulation of chemotherapy response of cancer cells has been demonstrated to be dynamic and reversible.^10^ Numerous studies have shown that overcoming TRAIL resistance is possible by using secondary agents, such as the histone deacetylase inhibitors,^11, 12, 13^ corroborating the idea that the resistance can be reversed by epigenetic reprogramming.

The epigenome of cells are maintained by dynamic histone and DNA modifications throughout the chromatin by a group of chromatin-modifying enzymes (CMEs) called ‘writers’ such as histone acetyltransferases, histone methyltransferases (HMTs) and DNA methyltransferases (DNMTs).^14^ These modifications can be removed by ‘erasers’ such as histone deacetylases (HDACs) and histone demethylases (HDMs), or can be recognized by ‘readers’ such as bromodomain-containing proteins.^15^ Although the molecular mechanisms leading to aberrant cancer epigenomes are becoming better understood,^16, 17, 18^ most studies in GBM merely focus on DNA hyper/hypomethylation.^19^ Therefore, an unbiased and comprehensive assessment of roles of CMEs in apoptosis resistance is needed.

In this study, we aimed to interrogate the function of CMEs regulating apoptotic response in GBM and undertook a loss-of-function approach using short-hairpin RNAs (shRNAs) that were designed against a select but diverse set of CMEs and associated proteins. We have shown that loss of KDM2B – a H3K36-specific histone demethylase – primed GBM cells for apoptosis through the induction of proapoptotic genes and the suppression of antiapoptotic genes. Our results suggest KDM2B as a novel central epigenetic regulator of GBM cell apoptosis and identify it as a potential proapoptotic target for future of GBM treatment.

## Results

### Interrogation of the CMEs reveals KDM2B as a modulator of TRAIL-induced apoptosis in GBM cells

**Figure 1:**
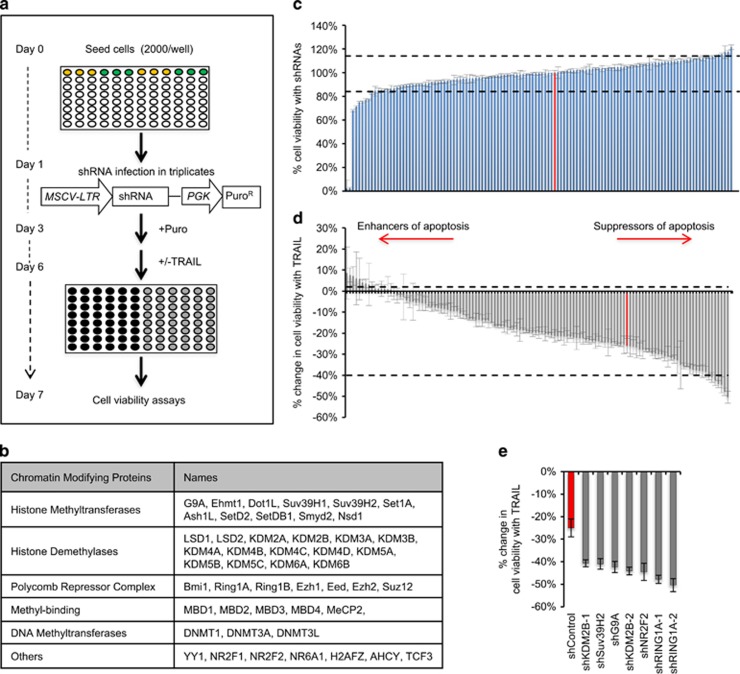
shRNA screen for TRAIL regulating CMEs in GBMs. (**a**) Schematic diagram of the experimental setup for the shRNA TRAIL screen in U87MG cells. Cells were individually infected with 125 different shRNAs targeting CMEs and selected with puromycin. Viability analysis was conducted after 100 ng/ml TRAIL treatment for 24 h. (**b**) Table of categorized 48 different CMEs targeted in the shRNA screen. (**c**) Percent viability analysis of the untreated cells after infection. (**d**) Percent change viability analyses of the infected and TRAIL-treated cells. (**e**) Top sensitizer hits below 2 S.D. Data were normalized to shControl cells. Red bar indicates shControl. Genes lying below and over that of shControl were classified as suppressors and enhancers of apoptosis, respectively. Dotted lines indicate 2 S.D. from % viability change of shControl cells

To identify the key CMEs regulating the apoptotic response of GBM cells, we applied a shRNA-based loss-of-function screen in U87MG cell line (Figure 1a). We used a library of 60 shRNAs published before,^20^ and expanded it to 125 shRNAs targeting 48 different CME genes with two or three separate shRNAs. The targeted CMEs included DNMTs, HMTs, HDMs, methylated DNA-binding proteins, polycomb-group proteins (PRCs) and a few transcription factors (Figure 1b). We first assessed the effects of shRNAs alone on U87MG cell viability, to identify shRNAs displaying intrinsic toxicity before TRAIL treatment. Two of the 125 shRNAs were eliminated from further analysis in the screen as they caused ~100% cell death after puromycin selection, possibly due to problems with viral packaging. Accordingly, 6 out of 125 shRNAs reduced cell viability more than 2 S.D. compared with shControl (Figure 1c). These were shRNAs targeting KDM5C (68±1%), KDM4C (71±1%), KDM4A (75±1%), KDM4A (75±1%), Set1A (77±1%) and KDM3B (77±1%). Five out of 125 shRNAs targeting MeCP2 (115±1%), MBD1 (115±1%), AHCY (117±5%), TCF3 (117±1%) and EZH1 (121±2%) caused minor increases in viability. However, the majority of the shRNAs did not significantly alter cell viability (Figure 1c). To identify shRNAs that modulated TRAIL sensitivity, we assessed the percent viability changes after TRAIL treatment. Accordingly, we categorized CMEs into two groups based on the phenotype observed upon their silencing compared with shControl cells: (1) suppressors of apoptosis, if their knockdown sensitized the cells to TRAIL, (2) enhancers of apoptosis, if their knockdown conferred cells more TRAIL resistant (Figure 1d). While TRAIL caused 25±4% reduction in the viability of shControl cells, there were seven shRNAs that sensitized cells to TRAIL. These were targeting five genes, namely *Suv39H2* (41±2%), *G9A* (42±2%), *NR2F2* (45±4%), *RING1A* (48±2 or 50±3%) and *KDM2B* (41±2 or 44±2%) (Figure 1e). We then focused on the genes *RING1A* and *KDM2B*, whose knockdown led to a phenotype with two independent shRNAs (Figure 1e). Taken together, our screen identified KDM2B and RING1, an H3K36-specific demethylase and an E3 ubiquitin-protein ligase of H2AK119, respectively, as novel regulators of TRAIL response.

### Loss of KDM2B cooperates with TRAIL to reduce GBM cell viability

**Figure 2:**
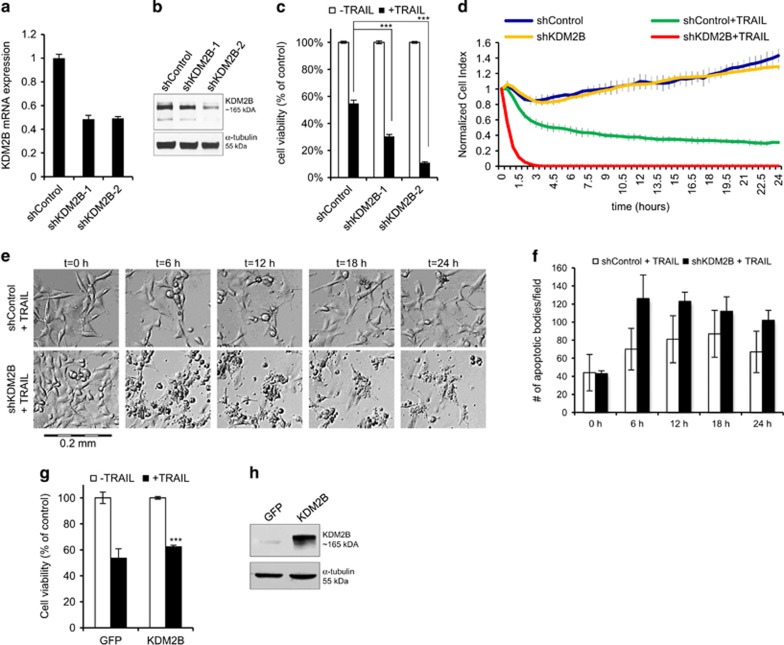
Loss of KDM2B sensitizes GBM cells to TRAIL. (**a**) Expression analysis of KDM2B in shControl and shKDM2B GBM cells. Expression levels were normalized to shControl cells. (**b**) Western blot analysis of GBM cells expressing either control shRNA or one of shRNAs targeting KDM2B. (**c**) Viability analyses of GBM cells showing reduced viability against TRAIL upon KDM2B knockdown. (**d**) Real-time viability analysis of shControl and shKDM2B cells upon TRAIL treatment for 24 h. Cell indices were normalized to time of TRAIL addition. (**e**) Representative images of shControl and shKDM2B cells treated with TRAIL for 24 h. Images were taken from same coordinates by inverted live-cell light microscope (× 10 magnification). (**f**) Plot displaying numbers of apoptotic bodies per frame in shControl and shKDM2B fields, quantified using ImageJ (*n*=3 different fields) (*, ** and *** denotes *P*<0.05, *P*<0.01 and *P*<0.001, two-tailed Student’s *t*-test). All experiments were conducted with U87MG cells with 100 ng/ml TRAIL treatment. (**g**) Viability analyses of GBM cells showing less TRAIL response upon KDM2B overexpression. (**h**) Western blot analysis of GBM cells overexpressing control (pBabeGFP) or KDM2B (pBabeKDM2B)

To assess the function of the novel apoptosis-modulating CMEs we identified in GBMs, we first checked the knockdown efficiency of the shRNAs and observed silencing down to 17–40% (Supplementary Figure 1). We then focused on KDM2B, as its protumorigenic functions have been demonstrated in various solid and hematological malignancies;^21, 22^ however, its function in GBMs was not defined. Quantitative RT-PCR (qRT-PCR) analysis of *KDM2B* levels revealed that both shRNAs reduced *KDM2B* mRNA levels down to ~50% (Figure 2a). However, shKDM2B-2 led to a more robust reduction at protein levels (Figure 2b). To assess whether the shRNAs targeting KDM2B is specific, we checked the levels of other KDM family members upon KDM2B knockdown and observed no major alterations in their levels (Supplementary Figure 2).

To further validate the screen results, we conducted ATP-based cell viability analysis of cells transduced with both shKDM2B vectors and verified that cells with reduced KDM2B exhibit cell death significantly more than the controls (Figure 2c). To examine these differences in cell death further, we used an assay that measures cell growth in real time, where cells’ electrical impedance in a well is measured and transformed into a cell index. Accordingly, silencing of KDM2B not only augmented TRAIL response but also accelerated the process of cell death (Figure 2d). There was no observable difference between untreated cells, showing that KDM2B knockdown has no significant effects on short-term (24 h) proliferation dynamics. This phenotype was also validated by live-cell imaging, where the morphology of individual cells, as well as cell death processes, was observed in real time (Figure 2e and Supplementary Videos 1–4). As shown by automated quantification of cellular blebs that were indicative of apoptotic bodies, the process of apoptosis was accelerated in shKDM2B cells compared with controls (Figure 2f). The number of apoptotic bodies per frame reached its maxima within 6 h of TRAIL treatment in shKDM2B cells, earlier and significantly in higher numbers than shControl cells. To examine whether KDM2B effects can be recapitulated in additional GBM cell lines, we used a more TRAIL-sensitive line, T98G in parallel. There, KDM2B knockdown led to increased TRAIL sensitivity, in accordance with U87MG cells (Supplementary Figure 3).

Next, as a complementary approach, we tested whether overexpression of KDM2B would result in an opposite response for TRAIL. Accordingly, KDM2B overexpression led to a modest but significant reduction in TRAIL response in U87MG cells (Figures 2g, h and Supplementary Figure 4). KDM2B overexpression conferred T98G cells more resistant to TRAIL as well (Supplementary Figure 5). Taken together, our data suggest that KDM2B expression suppresses TRAIL response and therefore its inhibition leads to increased TRAIL sensitivity in GBM cells.

### Silencing of KDM2B sensitizes GBM cells to TRAIL-induced apoptosis

**Figure 3:**
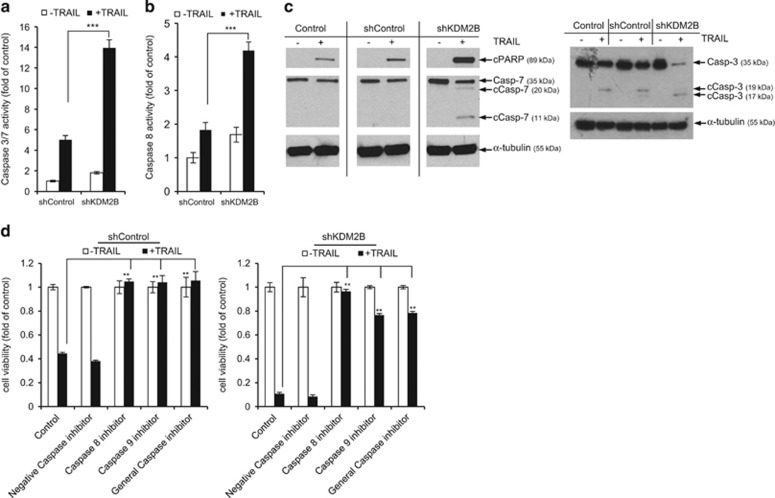
GBM cells display augmented apoptotic response upon KDM2B silencing. (**a**) Caspase-3/7 and (**b**) caspase-8 activity analyses of shControl and shKDM2B cells after 3 h treatment with TRAIL. Data were normalized to untreated shControl cells. (**c**) Western blot analyses of control, shControl and shKDM2B cells for cleaved PARP, caspase-3 and caspase-7 after 6 h treatment with TRAIL. (**d**) Cell viability analyses of shControl and shKDM2B cells cotreated with TRAIL and caspase-8, caspase-9 or general caspase inhibitors. Data were normalized to untreated cells of each condition (*, ** and *** denotes *P*<0.05, *P*<0.01 and *P*<0.001, two-tailed Student’s *t*-test). All experiments were conducted with U87MG cells with 100 ng/ml TRAIL treatment

To better understand how downregulation of KDM2B reduces viability and increases TRAIL response, we examined the components of the apoptotic machinery in shControl and shKDM2B cells. We observed a 3-fold increase in caspase-3/7 and a 2.5-fold increase in caspase-8 activity levels (Figures 3a and b). Analysis of the whole-cell lysates of shControl and shKDM2B cells revealed that caspase-7 is indeed upregulated in shKDM2B cells at the protein level (Figure 3c). Importantly, we observed that there is a significant increase in levels of cleaved PARP, caspase-3 and caspase-7, all of which are indicators of apoptotic cell death. We have also observed a very slight increase in cleaved Bid levels in shKDM2B cells upon TRAIL treatment (Supplementary Figure 6).

To test the functional roles of caspases in shKDM2B-mediated apoptotic sensitization, we used specific inhibitors for caspase-8, -10 (Z-IETD-FMK) and caspase-9 (Z-LEHD-FMK). In addition, we used a general caspase inhibitor that target caspase-8, -10, -9, -3 and -7 (Z-VAD-FMK), and an inactive caspase inhibitor (Z-FA-FMK) as control. In both shControl and shKDM2B cells, inhibition of all caspases abolished the TRAIL response, suggesting that the observed augmented apoptotic response in shKDM2B cells was dependent on both intrinsic and extrinsic apoptosis pathways (Figure 3d).^23^

To examine whether KDM2B modulates the response of GBM cells to proapoptotic agents that trigger intrinsic apoptosis, besides the extrinsic apoptosis that is triggered by TRAIL, we conducted cell viability experiments with Bcl-2/Bcl-XL inhibitors, ABT-263 and ABT-737 (Supplementary Figures 7 and 8). While ABT-263 or ABT-737 alone did not markedly decrease cell viability, combination of ABT-263 or ABT-737 with TRAIL did. We observed that knockdown or overexpression of KDM2B in U87MG cells did not affect this cell line’s overall response to ABT-263 and ABT-737 individual treatments. However, knockdown of KDM2B sensitized these cells to ABT and TRAIL combination (Supplementary Figure 7), and KDM2B overexpression was able to confer GBM cells more resistant to ABT and TRAIL combination (Supplementary Figure 8). These results suggested that the interplay between KDM2B and apoptosis is likely to be valid for both the intrinsic and extrinsic arms of apoptosis.

### Silencing of KDM2B leads to deregulation of apoptosis-related genes

**Figure 4:**
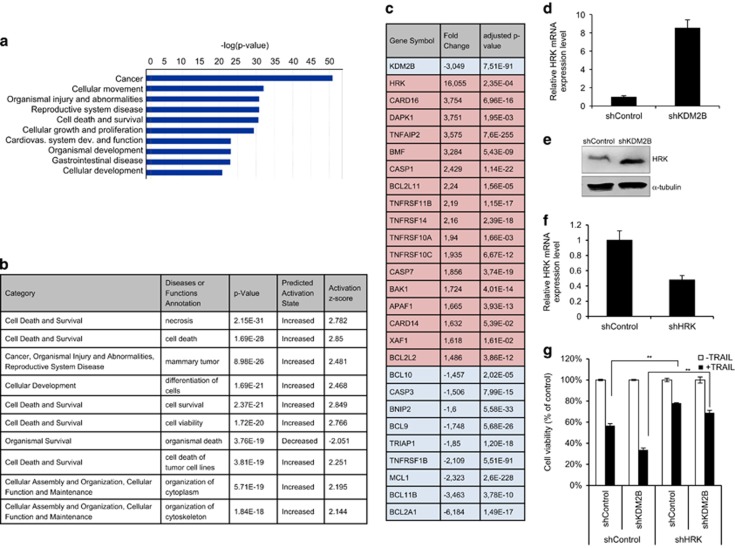
KDM2B regulates apoptotic machinery at transcriptional level in GBM cells. (**a**) Ingenuity pathway analysis of RNA-seq results. (**b**) List of the top 10 canonical pathways that were most significantly upregulated or downregulated upon KDM2B loss. (**c**) Custom list of genes displaying top deregulated apoptosis-related genes along with transcript level of KDM2B (*n*=2 biological replicates, average number of reads of shKDM2B cells were normalized to shControl cells). (**d**) qRT-PCR validation of HRK gene expression levels of shKDM2B cells. Expression levels were normalized to shControl. (**e**) Western blots showing HRK upregulation in shKDM2B cells at the protein level (**f**) qRT-PCR quantification of HRK mRNA level of HRK knockdown cells. Expression levels were normalized to shControl. (**g**) Cell viability analysis upon TRAIL treatment of shControl and shHRK cells transduced with either shControl or shKDM2B. Data were normalized to untreated cells of each group. (*,** and *** denotes *P*<0.05, *P*<0.01 and *P*<0.001, two-tailed Student’s *t*-test). All experiments were conducted with U87MG cells with 100 ng/ml TRAIL treatment

KDM2B can bind throughout the genome by its CxxC-binding domain,^24^ and its catalytic demethylase activity on the H3K36me2/me3 residues is associated with gene repression. However, the genes that are regulated in favor of apoptosis in GBM cells are unknown. To this end, we conducted RNA-sequencing (RNA-seq) analysis on shControl and shKDM2B cells. *KDM2B* expression levels were downregulated in shKDM2B cells compared with shControl cells −3.049-fold (adjusted *P*-value 7.51E−91), attesting to the validity of RNA-seq experiments. In total, 2457 genes were differentially expressed between shControl and shKDM2B cells. Ingenuity pathway analysis of these genes revealed that ‘cell death and survival’ and ‘cellular growth and proliferation’ pathways were among the top 10 that were significantly different between shControl and shKDM2B cells with –log *P*-values of 25–30 (Figure 4a). Five out of the top 10 altered functions were categorized as ‘cell death and survival’ where these gene sets were predicted to be activated with activation *z*-scores of >2.5 (Figure 4b). In addition, top 2 of these 10 categories were ‘cell death and survival’, suggesting that KDM2B silencing alters cell death- and survival-associated genes in GBM cells.

We were particularly interested in the cell death-related pathway in the shKDM2B cells, and therefore focused our attention on individual differentially expressed genes and generated a custom list of apoptosis-related genes that, when deregulated, can affect the outcome of TRAIL-induced apoptosis (Figure 4c). When we sorted the list of apoptosis-related genes based on differential expression, the most significant change was on *HRK* gene, with striking 16-fold induction in shKDM2B cells compared with shControl cells. The expression of other key apoptosis players, including *CASP7, DAPK1, BAK1* and *TNFRSF10A (DR4)* were also induced between 1.5- to 3.7-fold (Figure 4c). To validate the RNA-seq results, we designed gene-specific primers (Supplementary Table 1), performed qRT-PCR and confirmed the differential expression of *HRK* (Figure 4d). Further, qRT-PCR analyses of apoptosis-related genes showed proapoptotic *DAPK1, CARD16, TNFAIP2, TNFRSF10c, BCL2L11, BMF, XAF1, TNFRSF14, TNFRSF11B, APAF1, CASP1, CASP7, BCL2L2* and *BAK1* were induced, while antiapoptotic *MCL1, BCL11B* and *BCL2A1* were repressed (Supplementary Figure 9). These results suggest that silencing KDM2B causes genome-wide transcriptional changes and specifically alters apoptotic machinery in favor of apoptosis in GBM cells.

Because *HRK* was the top induced gene in shKDM2B cells, we further tested its functional association with KDM2B. HRK is a BH3-only Bcl-2 family member that can antagonize antiapoptotic proteins Bcl-2 and Bcl-xL.^25^ While *HRK*’s role is mostly studied in nervous system,^26, 27^ its role in cancers, especially in GBM is not well defined.^25^ To assess the increased HRK expression upon KDM2B loss, we performed western blotting and observed increased HRK protein levels in shKDM2B cells compared with controls (Figure 4e). To then test the role of HRK, we used shRNA targeting *HRK* and observed significant reduction in its mRNA expression (Figure 4f). Silencing of HRK led to significant but partial recovery from TRAIL-induced death in both shControl and shKDM2B cells (Figure 4g). These results suggest that *HRK* is potentially a downstream gene regulated by KDM2B and may facilitate TRAIL response in GBM cells.

### Silencing of H3K36-specific HMTs leads to the opposite phenotype of KDM2B silencing in GBM cell apoptosis

**Figure 5:**
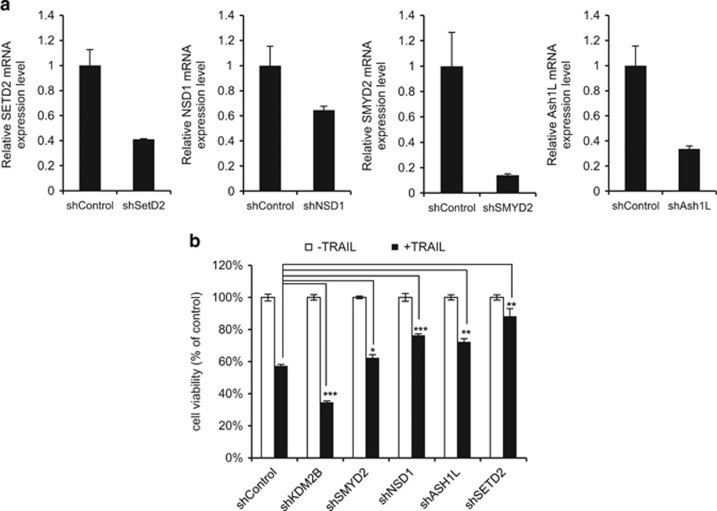
Loss of H3K36 HMTs confer GBM cells resistant to TRAIL reduced viability. (**a**) qRT-PCR analysis of mRNA levels of SETD2, NSD1, SMYD2 and ASH1L in transduced U87MG cells. Expression levels were normalized to shControl. (**b**) Viability analysis of U87MG cells expressing shRNAs targeting SMYD2, NSD1, ASH1L and SETD2 upon 100 ng/ml TRAIL treatment. Data were normalized to untreated condition of each group (*,** and *** denotes *P*<0.05, *P*<0.01 and *P*<0.001, two-tailed Student’s *t*-test)

H3K36-specific HMTs that catalyze the opposite function of KDM2B are known.^28^ Our shRNA library included four of these HMTs, namely SETD2, NSD1, ASH1L and SMYD2. As they carry out the opposite enzymatic activity of KDM2B, we assessed their effects on apoptotic response. We observed significant reduction in gene expression with each corresponding shRNA (Figure 5a). Individual knockdown of each enzyme did not lead to global differences in H3K36me2 or H3K36me3 levels as assessed by western blotting of histone extracts (Supplementary Figure 10), suggesting that H3K36me regulation at specific loci might be responsible for the observed phenotypic changes. While silencing of KDM2B followed by TRAIL treatment led to increased cell death, silencing of SETD2, NSD1, ASH1L, and SMYD2 individually led to significantly reduced cell death (Figure 5b). These results suggest that H3K36 methylation and demethylation at specific loci of apoptosis-related genes potentially regulate the apoptotic response of GBM cells to TRAIL.

### Loss of KDM2B attenuates tumor growth *in vivo*

**Figure 6:**
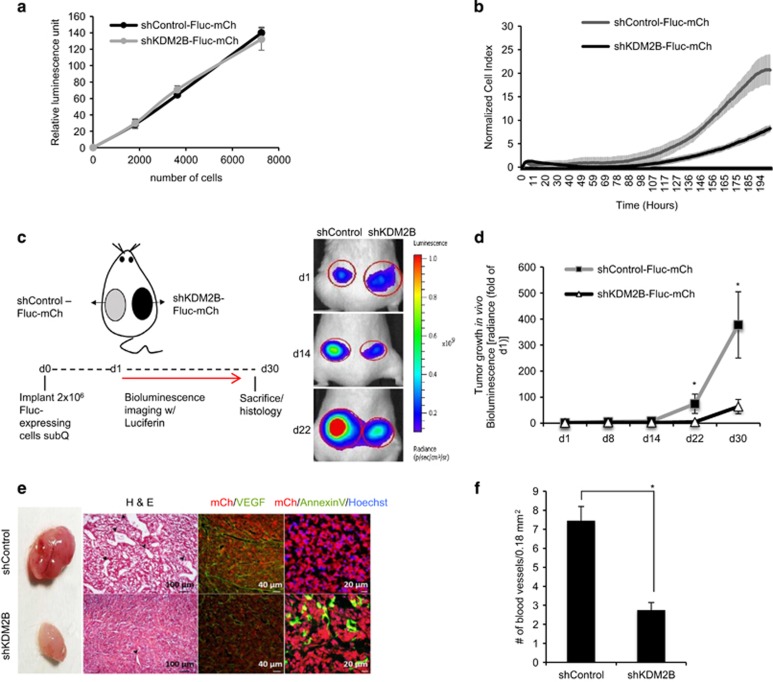
Knockdown of KDM2B attenuates tumor growth *in vivo*. (**a**) Plot demonstrating *in vitro* bioluminescence of shControl and shKDM2B cells transduced with Fluc-mCh constructs. (**b**) Long-term growth dynamic of shControl and shKDM2B cells were assessed by real-time cell analyzer for 202 h (*n*=6 wells for each group). (**c**) shControl-Fluc-mCh and shKDM2B-Fluc-mCh cells were subcutaneously implanted into nonobese diabetic/severe combined immunodeficiency (NOD/SCID) mice and assessed for tumor growth for 30 days. Representative images of bilateral tumors of same mice from days 1, 14 and 22 displaying normalized bioluminescent efficiencies acquired (blue to red indicates lower to higher radiance as photons/s/cm^2^/steradian). (**d**) Tumor growth was measured by bioluminescent radiance on five time points for 30 days. Data were normalized to day 1 signal of each group (*n*=7 tumors per group). (**e**) Representative histology and immunofluorescence sections of tumors stained with H&E, anti-VEGF and annexin-V. (**f**) Number of blood vessels per 18 mm^2^ are counted and plotted (*n*=3 fields per tumors)

To examine the effects of KDM2B loss on tumor growth *in vivo*, we generated shControl and shKDM2B cells expressing both firefly luciferase (Fluc) and mCherry. The bioluminescence signals of shControl-Fluc-mCh and shKDM2B-Fluc-mCh cells were comparable *in vitro* (Figure 6a). To assess the effect of KDM2B loss on long-term GBM growth before *in vivo* implantation, we performed real-time cell analysis of shControl-Fluc-mCh and shKDM2B-Fluc-mCh cells for 200 h. We observed that knockdown of KDM2B alone reduces the proliferation rate of GBM cells in long term (Figure 6b), while within short term of 24 h the proliferation rate is comparable with control cells, confirming our previous observation (Figure 2d). Long-term analysis of tumor growth for 30 days with noninvasive bioluminescence imaging revealed that loss of KDM2B also attenuates tumor growth *in vivo* (Figures 6c and d). Immunofluorescence and histological analysis of the tumors showed that loss of KDM2B led to reduced angiogenic capacity of the tumors as assessed by regular H&E and VEGF staining. Moreover, there were significantly more annexin-V-positive tumor cells within shKDM2B tumors compared with controls (Figures 6e and f). Taken together, these results suggest that KDM2B silencing led to decreased tumor growth in long term, which may be attributable to the increased basal levels of apoptosis.

## Discussion

In this study, we have used an unbiased shRNA screen to show that the deregulation of a CME in a GBM cell line can cause sensitization to TRAIL through induction of proapoptotic machinery. Several lines of evidence support this argument. First, loss of KDM2B cooperated with TRAIL to reduce GBM cell viability and augmented the apoptotic response as assessed by the hallmarks of apoptosis upon TRAIL treatment. Further, whole-genome transcriptome analysis suggested that endogenous levels of KDM2B in GBM is correlated with suppressed state of proapoptotic machinery, whose knockdown results in induction of apoptosis by inducing proapoptotic genes and repressing antiapoptotic genes. *HRK* was the top induced gene upon KDM2B loss and its silencing partially recovered the observed sensitization phenotype, suggesting that *HRK* might be a novel and direct target of KDM2B-mediated apoptosis regulation in GBM cells.

In our screen, the majority of the shRNAs (110/125) targeting CMEs did not affect TRAIL response on their own. However, some were able to prime GBM cells for apoptosis. Although our shRNA screen targeted diverse types of CMEs, such as writers (DNMTs and HMTs), erasers (HDMs), readers (methyl-DNA-binding proteins) and other chromatin regulatory proteins such as polycomb-group proteins (PRCs), it did not cover all types and members of CMEs. For example, HATs and HDACs were not included in the shRNA screen. It will be of interest to see whether shRNA-mediated knockdown of HDACs would prime tumor cells for apoptosis as their small drug inhibition were previously reported to be implicated in TRAIL sensitization.^29^

One major hit from our screen was KDM2B. KDM2B is a H3K36me2 demethylase that is indicated as a regulator of cell growth.^22, 30, 31^ KDM2B, by its CxxC-ZF domain, can bind to CpG islands and can catalyze demethylation of H3K36 or H3K4 residues through its JmjC domain.^24^ KDM2B has been shown to be required for the tumorigenesis of acute myeloid leukemia, where it acts as an oncogene through silencing *p15/Ink4b*.^32^ Similarly, KDM2B has been shown to regulate self-renewal capacity of cancer and cancer stem cells in hematological and pancreatic cancers, and its expression is positively correlated with advanced tumor grade.^21, 22^ A recent study displayed the protumorigenic role of KDM2B in a broad range of human tumors.^33^ To our knowledge, KDM2B has not been studied previously in brain malignancies and the present work is the first study to display its novel oncogenic role in GBMs.

Interestingly, we found that shRNAs targeting the H3K36-specific HMTs, namely SETD2, NSD1, SMYD2, ASH1L, known to conduct the opposite enzymatic activity of KDM2B by catalyzing the addition of methyl groups to H3K36 residues, conferred GBM cells partly resistant to TRAIL-induced apoptosis. Therefore, we believe that H3K36 methylation can be a central regulator of TRAIL-induced apoptosis in GBM cells, and it would be interesting to dissect out the exact molecular mechanism by testing the function of HMTs in combination, and conducting chromatin analysis in the future. We show that short-term KDM2B loss by itself does not cause major changes in cell survival; however, long-term effect of KDM2B suppression is observed in our *in vivo* xenograft models. This suggests that while short-term loss of KDM2B can alter the epigenetic landscape of tumor cells in favor of apoptosis, long-term consequences of the epigenome alterations can reduce proliferation and be detrimental without additional external apoptotic stimuli. Therefore, it will be important to uncouple the exact mechanisms of how KDM2B regulates proliferation rate, survival and apoptosis in GBM cells. In this study, we focused on the examination of apoptotic regulation and showed that KDM2B can alter apoptotic response of GBM cells to TRAIL-induced apoptosis. This is consistent with a previous study showing that KDM2B regulated the apoptotic response of pancreatic cancer cells.^34^ Our consistent results with multiple GBM cell lines and multiple proapoptotic agents such as TRAIL and BH3 mimetics suggest that KDM2B could be a global regulator of cell death in GBM cells.

How KDM2B might regulate apoptotic priming in GBM cells was also examined in this study by comparing the transcription profiles of shControl and shKDM2B cells. Accordingly, among many apoptosis-related genes taking place in TRAIL pathway, proapoptotic genes such as *DR4* and executioner *caspase-7* were induced. Interestingly, *HRK*, a BH3-only Bcl-2 family member, that can antagonize antiapoptotic proteins Bcl-2 and Bcl-xL,^25^ was the top induced gene. As such, silencing of *HRK* partly recovered the apoptotic sensitization, suggesting that *HRK* is directly linked to KDM2B-mediated regulation. There were other genes that were modulated by KDM2B loss, such as *DAPK1* or *BMF*, which could also be partly responsible for apoptotic sensitization. DAPK1 is a proapoptotic tumor suppressor that has been shown to be inactivated in various cancer settings,^35, 36^ whereas antiapoptotic BH3-only BMF can also sensitize cells via directly interacting with Bcl-2, Bcl-xL, Bcl-w and Mcl-1.^37^ On the other hand, knockdown of KDM2B led to suppression of antiapoptotic genes *MCL1*, *BCL11B* and *BCL2A1*. This could be through an indirect cascade of events or a direct consequence of partial loss of KDM2B.^22^ Interestingly, BCL11B silencing has been demonstrated to induce apoptosis in malignancies.^38^ BCL2A1 is overexpressed in various cancers and its upregulation is characterized as a major resistance factor against proapoptotic therapy in cancer.^39^ MCL1 is one of the key gatekeepers regulating apoptosis and has been targeted by small drug inhibitors to sensitize cancer cells.^40, 41^ Further research is warranted to understand how KDM2B regulates the expression of these apoptosis regulators at the chromatin level.

**Figure 7:**
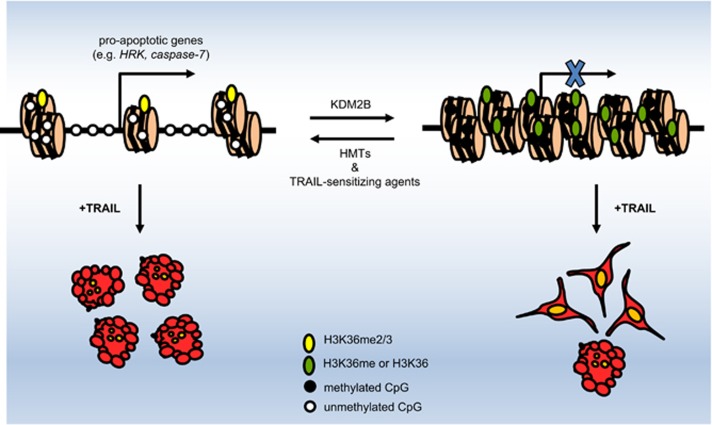
A possible mechanism of KDM2B regulation of apoptotic response in GBM cells. KDM2B modulates the H3K36 methylation levels of target proapoptotic genes to suppress their expression levels. RNAi-mediated knockdown or TRAIL-sensitizing agents downregulate KDM2B, which in turn lead to sensitization of GBM cells by increased transcription of proapoptotic genes. In accordance, silencing of HMTs responsible for the same histone modification confers GBM cells more resistant to TRAIL-induced apoptosis

Taken together, we show that KDM2B regulates the apoptotic response of GBM cells by turning on the apoptotic machinery that is otherwise dormant. Therefore, combining epigenetic reprogramming drugs with standard chemotherapeutics or with proapoptotic therapies are promising therapeutic approaches. To this end, previously, several agents have been combined with TRAIL, including HDAC inhibitors. Interestingly, bortezomib,^42^ MS-275,^11^ and a recent drug identified by our group, mitoxantrone,^43^ have all suppressed the expression levels of KDM2B in favor of cell death (Supplementary Figure 11). Therefore, it is plausible to suggest that regulators of apoptosis might be acting through regulating the central epigenetic machinery in favor of enhanced cell death (Figure 7).

GBM is a complex cancer with extremely poor prognosis and limited therapeutic options. Therefore, designing new and effective approaches for GBM is necessary. Drugs targeting CMEs are emerging as anticancer therapeutics.^44^ Our results corroborate the notion that combinatorial uses of epigenetic drugs with proapoptotic agents hold great promise. To this end, specific chemical inhibitors for CMEs such as a KDM2B inhibitor can yield effective combination therapies for subgroups of GBM patients.

## Materials and methods

### Cell lines

U87MG GBM cells and 293T cells were purchased and authenticated from ATCC (Manassas, VA, USA). All cells were cultured in DMEM medium supplemented with 10% FBS and 1% penicillin–streptomycin (Gibco, Grand Island, NY, USA) at 37 °C in a humidified chamber with 5% CO_2_ as described.^11^ Puromycin selection was applied at a final concentration of 1 *μ*g/ml for 3–7 days.

### Reagents

TRAIL was commercially supplied (Enzo Life Sciences, Farmingdale, NY, USA) or produced from 293T cells as described.^45^ Briefly, the extracellular portion of human TRAIL (amino acids 95–281) was cloned into pBabe retroviral expression vectors to produce functionally active TRAIL,^46^ which was quantified by TRAIL ELISA according to the manufacturer’s instructions (Abcam, Cambridge, UK). Caspase inhibitors (BD Pharmingen, San Diego, CA, USA) were as follows: Z-IETD-FMK (caspase-8 inhibitor), Z-VAD-FMK (general caspase inhibitor), Z-FA-FMK (negative control), Z-LEHD-FMK (caspase-9 inhibitor) and Ac-DEVD-CHO (caspase-3/7 inhibitor). Bcl-2, Bcl-xL inhibitors ABT-263 and ABT-737 were purchased from Cayman Chemicals (Ann Arbor, MI, USA)

### Generation of the shRNA library

One hundred and twenty-five of total shRNAs targeting chromatin-modifying proteins and HRK were designed using the RNAi Codex program. Accordingly, two to three shRNAs were designed using 97-mer oligos and cloning into pSMP vector system. The designed oligos were PCR amplified using the following primer pairs: (forward: 5′-GATGGCTGCTCGAGAAGGTATATTGCTGTTGACAGTGAGCG-3′ reverse: 5′-GTCTAGAGGAATTCCGAGGCAGTAGGC-3′). PCR products were gel purified, digested with *Eco*RI and *Xho*I and ligated into the retroviral pSMP vector as described,^20^ and verified by sequencing. shRNA targeting the Fluc was used as shControl. The sequences of selected shRNAs are listed in Supplementary Table 2; viral packaging was conducted as described.^20^ The 293T cells were seeded as 2.5 × 10^6^ cells per plate and transfected with shRNA, VSV-G and pUMVC plasmids with Fugene transfection reagent (Promega, Madison, WI, USA). The virus containing media was collected, filtered through 0.45 *μ*m filters (Millipore, Billerica, MA, USA) and stored at −80 °C.

### shRNA screen

U87MG cells were seeded as 2500 cells per well into black 96-well plates. Next day, cells were infected with 80 *μ*l of viral supernatants with protamine sulfate (10 *μ*g/ml) in triplicates. After 2 days, puromycin (1 *μ*g/ml) was added for 3 days. Following selection, cells were treated with TRAIL (100 ng/ml) or control solution for 24 h, followed by cell viability assays. shRNAs that caused reduction in cell viability, as well as shRNAs that augmented TRAIL response in at least two independent experiments, were identified and further characterized.

### Cloning

The 4617 bp ORF of KDM2B in pCAG-puro, including 2x Strep-tag II and 1x FLAG-tag at its N terminus, was cloned by the flanking *Eco*RI cut sites into retroviral pBabe-puro mammalian expression vector.

### Cell viability and caspase activity assays

Viability of the cells was measured using ATP-based Cell Titer Glo assays (Promega) according to the manufacturer’s instructions using a plate reader (BioTek’s Synergy H1, Winooski, VT, USA). All experiments were performed in triplicates. To further validate KDM2B phenotype, stable cell lines of shKDM2B or shControl cells were expanded for three to four passages. Accordingly, 10 000 cells per well were seeded in triplicates and treated with TRAIL (0–100 ng/ml) for 24 h. To test caspase inhibition, each caspase inhibitor was used at 20 *μ*M final concentration; cells were cotreated with inhibitors and TRAIL. For caspase activity measurements, cells were seeded in 96-well plates as 10 000 cells per well in triplicates, treated with TRAIL (0–100 ng/ml) for 3 h and caspase-8 and -3/7 activities were measured using Caspase Glo (Promega) assays according to the manufacturer’s instructions.

### Real-time cell growth analysis

xCELLigence RTCA SP Station and Analyzer (ACEA Biosciences, San Diego, CA, USA) was used for real-time cell growth analysis. The 1000–5000 U87MG cells per well were seeded, and impedance of the wells were measured with 20 min intervals for 24 or 202 h. The amount of cell growth was analyzed and plotted using the RTCA Software (ACEA Biosciences).

### Live-cell microscopy

All live-cell imaging experiments were carried out by Olympus Xcellence Pro inverted microscope (Center Valley, PA, USA) with a × 10 air objective in a chamber at 37 °C, supplied with 5% CO_2_. Time-lapse images were captured right after TRAIL treatment for 24 h as 1440 frames per minute. Five random positions were recorded per sample per well. The number of apoptotic bodies was counted on each window using the ImageJ Software (NIH Image, Bethesda, MD, USA). Particles having size (pixel^2) of 10 to infinity and circularity of 0.60–1.00 were denoted as apoptotic bodies on each frame.

### qRT-PCR analysis

RNA isolation and cDNA synthesis were performed as described.^43^ qRT-PCRs was carried out using the primers in Supplementary Table 1. Details of qRT-PCR are given in Supplementary Information.

### Cell lysate preparation, histone extraction and immunoblotting

Cell lysate preparations were carried out as described previously.^43^ Details of histone extraction and immunoblotting are given in Supplementary Information.

### *In vivo* experiments

All *in vivo* experiments were approved by the institution review boards of Koç University (HADYEK no. 2014-22). U87MG cells stably expressing shControl or shKDM2B were used for *in vivo* experiments. Cells were supertransduced with lentiviral vectors encoding Fluc and mCherry as described.^46^ mCherry expression was verified with red fluorescence, and Fluc activity was verified with *in vitro* luminescence assays. Accordingly, increasing number of cells (0–50 000/well) were seeded on 96-well plates and incubated with d-luciferin (10 *μ*g/ml) for 10 min. Bioluminescence was measured by BioTek’s Synergy H1 plate reader.

A 6–8-week-old SCID mice were used for tumor implantation. A total of 2.5 × 10^6^ cells that stably expressed Fluc and mCherry were injected in 50 *μ*l PBS subcutaneously into seven SCID mice. One day after injection, tumor cells were visualized by Fluc imaging using IVIS Lumina III equipment (Perkin-Elmer, Rodgau, Germany) by injecting mice with 150 *μ*g/g body weight of d-luciferin (Biotium, Fremont, CA, USA) intraperitoneally. Tumor progression was monitored with sequential Fluc bioluminescence activity over a period of 1 month. All imaging experiments were performed under isoflurane anesthesia. Following data acquisition, mean, standard deviation and sum of the photon counts in the regions of interest were calculated and plotted. At the end, the tumors were dissected and analyzed with immunohistochemistry.

### Immunohistochemistry

Sections of tumors were analyzed by immunohistochemistry as described.^46^ Details are given in Supplementary Information.

### RNA-seq and analysis

Total RNAs of shControl and shKDM2B cells were isolated, RNA-seq library for each sample was prepared based on protocols on Illumina HiSeq 2500 to generate 50 bp single-end reads. The data discussed in this publication have been deposited in NCBI's Gene Expression Omnibus,^47^ and are accessible through GEO series accession number GSE81043 (https://www.ncbi.nlm.nih.gov/geo/query/acc.cgi?acc=GSE81043). Differentially expressed genes between shKDM2B and shControl were detected using DESeq,^48^ the complex biological processes induced by shKDM2B as compared with shControl were examined in the context of functional groups with Ingenuity Pathways Analysis, a web-delivered commercial application. Details of library preparation, statistical and functional analysis of differentially expressed genes are explained in Supplementary Information.

### Statistical analysis

Data were analyzed by Student's *t*-test when comparing two groups. Data were plotted as mean±s.e.m. and *P*-values were calculated. *, ** and *** denotes *P*<0.05, *P*<0.01 and *P*<0.001 on the figures.

## References

[bib1] Holland EC. Glioblastoma multiforme: the terminator. Proc Natl Acad Sci USA 2000; 97: 6242–6244. 10841526 10.1073/pnas.97.12.6242 PMC33993

[bib2] Berens ME, Giese A. '...those left behind.' Biology and oncology of invasive glioma cells. Neoplasia 1999; 1: 208–219. 10935475 10.1038/sj.neo.7900034 PMC1508082

[bib3] Fulda S. Tumor resistance to apoptosis. Int J Cancer 2009; 124: 511–515. 19003982 10.1002/ijc.24064

[bib4] Takeda K, Hayakawa Y, Smyth MJ, Kayagaki N, Yamaguchi N, Kakuta S et al. Involvement of tumor necrosis factor-related apoptosis-inducing ligand in surveillance of tumor metastasis by liver natural killer cells. Nat Med 2001; 7: 94–100. 11135622 10.1038/83416

[bib5] Lemke J, von Karstedt S, Zinngrebe J, Walczak H. Getting TRAIL back on track for cancer therapy. Cell Death Differ 2014; 21: 1350–1364. 24948009 10.1038/cdd.2014.81 PMC4131183

[bib6] Pan R, Hogdal LJ, Benito JM, Bucci D, Han L, Borthakur G et al. Selective BCL-2 inhibition by ABT-199 causes on-target cell death in acute myeloid leukemia. Cancer Discov 2014; 4: 362–375. 24346116 10.1158/2159-8290.CD-13-0609 PMC3975047

[bib7] Vaillant F, Merino D, Lee L, Breslin K, Pal B, Ritchie ME et al. Targeting BCL-2 with the BH3 mimetic ABT-199 in estrogen receptor-positive breast cancer. Cancer Cell 2013; 24: 120–129. 23845444 10.1016/j.ccr.2013.06.002

[bib8] Dimberg LY, Anderson CK, Camidge R, Behbakht K, Thorburn A, Ford HL. On the TRAIL to successful cancer therapy? Predicting and counteracting resistance against TRAIL-based therapeutics. Oncogene 2013; 32: 1341–1350. 22580613 10.1038/onc.2012.164 PMC4502956

[bib9] Elias A, Siegelin MD, Steinmüller A, von Deimling A, Lass U, Korn B et al. Epigenetic silencing of death receptor 4 mediates tumor necrosis factor-related apoptosis-inducing ligand resistance in gliomas. Clin Cancer Res 2009; 15: 5457–5465. 19706813 10.1158/1078-0432.CCR-09-1125

[bib10] Sharma SV, Lee DY, Li B, Quinlan MP, Takahashi F, Maheswaran S et al. A chromatin-mediated reversible drug-tolerant state in cancer cell subpopulations. Cell 2010; 141: 69–80. 20371346 10.1016/j.cell.2010.02.027 PMC2851638

[bib11] Bagci-Onder T, Agarwal A, Flusberg D, Wanningen S, Sorger P, Shah K. Real-time imaging of the dynamics of death receptors and therapeutics that overcome TRAIL resistance in tumors. Oncogene 2013; 32: 2818–2827. 22824792 10.1038/onc.2012.304 PMC3676868

[bib12] Lakshmikanthan V, Kaddour-Djebbar I, Lewis RW, Kumar MV. SAHA-sensitized prostate cancer cells to TNFα-related apoptosis-inducing ligand (TRAIL): mechanisms leading to synergistic apoptosis. Int J Cancer 2006; 119: 221–228. 16450389 10.1002/ijc.21824

[bib13] Aguilera DG, Das CM, Sinnappah-Kang ND, Joyce C, Taylor PH, Wen S et al. Reactivation of death receptor 4 (DR4) expression sensitizes medulloblastoma cell lines to TRAIL. J Neurooncol 2009; 93: 303–318. 19148581 10.1007/s11060-008-9788-x PMC3820292

[bib14] Falkenberg KJ, Johnstone RW. Histone deacetylases and their inhibitors in cancer, neurological diseases and immune disorders. Nat Rev Drug Discov 2014; 13: 673–691. 25131830 10.1038/nrd4360

[bib15] Rotili D, Mai A. Targeting histone demethylases: a new avenue for the fight against cancer. Genes Cancer 2011; 2: 663–679. 21941621 10.1177/1947601911417976 PMC3174264

[bib16] Rodríguez-Paredes M, Esteller M. Cancer epigenetics reaches mainstream oncology. Nat Med 2011; 17: 330–339. 21386836 10.1038/nm.2305

[bib17] Suvà ML, Riggi N, Bernstein BE. Epigenetic reprogramming in cancer. Science 2013; 339: 1567–1570. 23539597 10.1126/science.1230184 PMC3821556

[bib18] Nair SS, Kumar R. Chromatin remodeling in cancer: a gateway to regulate gene transcription. Mol Oncol 2012; 6: 611–619. 23127546 10.1016/j.molonc.2012.09.005 PMC3538127

[bib19] Maleszewska M, Kaminska B. Is glioblastoma an epigenetic malignancy? Cancers (Basel) 2013; 5: 1120–1139. 24202337 10.3390/cancers5031120 PMC3795382

[bib20] Onder TT, Kara N, Cherry A, Sinha AU, Zhu N, Bernt KM et al. Chromatin-modifying enzymes as modulators of reprogramming. Nature 2012; 483: 598–602. 22388813 10.1038/nature10953 PMC3501145

[bib21] Andricovich J, Kai Y, Peng W, Foudi A, Tzatsos A. Histone demethylase KDM2B regulates lineage commitment in normal and malignant hematopoiesis. J Clin Invest 2016; 126: 905–920. 26808549 10.1172/JCI84014 PMC4767361

[bib22] Tzatsos A, Paskaleva P, Ferrari F, Deshpande V, Stoykova S, Contino G et al. KDM2B promotes pancreatic cancer via polycomb-dependent and -independent transcriptional programs. J Clin Invest 2013; 123: 727–739. 23321669 10.1172/JCI64535 PMC3561797

[bib23] Fulda S, Debatin K-M. Extrinsic versus intrinsic apoptosis pathways in anticancer chemotherapy. Oncogene 2006; 25: 4798–4811. 16892092 10.1038/sj.onc.1209608

[bib24] Long HK, Blackledge NP, Klose RJ. ZF-CxxC domain-containing proteins, CpG islands and the chromatin connection. Biochem Soc Trans 2013; 41: 727–740. 23697932 10.1042/BST20130028 PMC3685328

[bib25] Inohara N, Ding L, Chen S, Núñez G. Harakiri, a novel regulator of cell death, encodes a protein that activates apoptosis and interacts selectively with survival-promoting proteins Bcl-2 and Bcl-X(L). EMBO J 1997; 16: 1686–1694. 9130713 10.1093/emboj/16.7.1686 PMC1169772

[bib26] Imaizumi K, Benito A, Kiryu-Seo S, Gonzalez V, Inohara N, Lieberman AP et al. Critical role for DP5/Harakiri, a Bcl-2 homology domain 3-only Bcl-2 family member, in axotomy-induced neuronal cell death. J Neurosci 2004; 24: 3721–3725. 15084651 10.1523/JNEUROSCI.5101-03.2004 PMC6729341

[bib27] Imaizumi K, Tsuda M, Imai Y, Wanaka A, Takagi T, Tohyama M. Molecular cloning of a novel polypeptide, DP5, induced during programmed neuronal death. J Biol Chem 1997; 272: 18842–18848. 9228060 10.1074/jbc.272.30.18842

[bib28] Wagner EJ, Carpenter PB. Manuscript A. Understanding the language of Lys36 methylation at histone H3. Nat Rev Mol Cell Biol 2012; 29: 997–1003. 10.1038/nrm3274 PMC3969746 22266761

[bib29] Nesterenko I, Wanningen S, Bagci-Onder T, Anderegg M, Shah K. Evaluating the effect of therapeutic stem cells on TRAIL resistant and sensitive medulloblastomas. PLoS ONE 2012; 7: e49219. 23145127 10.1371/journal.pone.0049219 PMC3492275

[bib30] Pfau R, Tzatsos A, Kampranis SC, Serebrennikova OB, Bear SE, Tsichlis PN. Members of a family of JmjC domain-containing oncoproteins immortalize embryonic fibroblasts via a JmjC domain-dependent process. Proc Natl Acad Sci USA 2008; 105: 1907–1912. 18250326 10.1073/pnas.0711865105 PMC2538857

[bib31] Liang G, He J, Zhang Y. Kdm2b promotes induced pluripotent stem cell generation by facilitating gene activation early in reprogramming. Nat Cell Biol 2012; 14: 457–466. 22522173 10.1038/ncb2483 PMC3544197

[bib32] He J, Nguyen AT, Zhang Y. KDM2b/JHDM1b, an H3K36me2-specific demethylase, is required for initiation and maintenance of acute myeloid leukemia. Blood 2011; 117: 3869–3880. 21310926 10.1182/blood-2010-10-312736 PMC3083299

[bib33] Kottakis F, Foltopoulou P, Sanidas I, Keller P, Wronski A, Dake BT et al. NDY1/KDM2B functions as a master regulator of polycomb complexes and controls self-renewal of breast cancer stem cells. Cancer Res 2014; 74: 3935–3946. 24853546 10.1158/0008-5472.CAN-13-2733 PMC4454481

[bib34] Ge R, Wang Z, Zeng Q, Xu X, Olumi AF. F-box protein 10, an NF-κB-dependent anti-apoptotic protein, regulates TRAIL-induced apoptosis through modulating c-Fos/c-FLIP pathway. Cell Death Differ 2011; 18: 1184–1195. 21252908 10.1038/cdd.2010.185 PMC3131965

[bib35] Xiong J, Li Y, Huang K, Lu M, Shi H, Ma L et al. Association between DAPK1 promoter methylation and cervical cancer: a meta-analysis. PLoS ONE 2014; 9: e107272. 25268905 10.1371/journal.pone.0107272 PMC4182030

[bib36] Ng HY, Wan TS, So CC, Chim CS. Epigenetic inactivation of DAPK1, p14ARF, mir-34a and -34b/c in acute promyelocytic leukaemia. J Clin Pathol 2014; 67: 626–631. 24811488 10.1136/jclinpath-2014-202276

[bib37] Delbridge ARD, Strasser A. The BCL-2 protein family, BH3-mimetics and cancer therapy. Cell Death Differ 2015; 22: 1071–1080. 25952548 10.1038/cdd.2015.50 PMC4572872

[bib38] Grabarczyk P, Przybylski GK, Depke M, Völker U, Bahr J, Assmus K et al. Inhibition of BCL11B expression leads to apoptosis of malignant but not normal mature T cells. Oncogene 2007; 26: 3797–3810. 17173069 10.1038/sj.onc.1210152

[bib39] Vogler M, Butterworth M, Majid A, Walewska RJ, Sun X-M, Dyer MJS et al. Concurrent up-regulation of BCL-XL and BCL2A1 induces approximately 1000-fold resistance to ABT-737 in chronic lymphocytic leukemia. Blood 2009; 113: 4403–4413. 19008458 10.1182/blood-2008-08-173310

[bib40] Gillissen B, Wendt J, Richter A, Richter A, Müer A, Overkamp T et al. Endogenous Bak inhibitors Mcl-1 and Bcl-xL: differential impact on TRAIL resistance in Bax-deficient carcinoma. J Cell Biol 2010; 188: 851–862. 20308427 10.1083/jcb.200912070 PMC2845080

[bib41] Leverson JD, Zhang H, Chen J, Tahir SK, Phillips DC, Xue J et al. Potent and selective small-molecule MCL-1 inhibitors demonstrate on-target cancer cell killing activity as single agents and in combination with ABT-263 (navitoclax). Cell Death Dis 2015; 6: e1590. 25590800 10.1038/cddis.2014.561 PMC4669759

[bib42] Unterkircher T, Cristofanon S, Vellanki SHK, Nonnenmacher L, Karpel-Massler G, Wirtz CR et al. Bortezomib primes glioblastoma, including glioblastoma stem cells, for TRAIL by increasing tBid stability and mitochondrial apoptosis. Clin Cancer Res 2011; 17: 4019–4030. 21525171 10.1158/1078-0432.CCR-11-0075

[bib43] Senbabaoglu F, Cingoz A, Kaya E, Kazancioglu S, Lack NA, Acilan C et al. Identification of mitoxantrone as a TRAIL-sensitizing agent for glioblastoma multiforme. Cancer Biol Ther 2016; 17: 546–557. 27029345 10.1080/15384047.2016.1167292 PMC4910918

[bib44] Mair B, Kubicek S, Nijman SMB. Exploiting epigenetic vulnerabilities for cancer therapeutics. Trends Pharmacol Sci 2014; 35: 136–145. 24529765 10.1016/j.tips.2014.01.001

[bib45] Hingtgen S, Ren X, Terwilliger E, Classon M, Weissleder R, Shah K. Targeting multiple pathways in gliomas with stem cell and viral delivered S-TRAIL and temozolomide. Mol Cancer Ther 2008; 7: 3575–3585. 19001440 10.1158/1535-7163.MCT-08-0640 PMC2748233

[bib46] Bagci-Onder T, Wakimoto H, Anderegg M, Cameron C, Shah K. A dual PI3K/mTOR inhibitor, PI-103, cooperates with stem cell-delivered TRAIL in experimental glioma models. Cancer Res 2011; 71: 154–163. 21084267 10.1158/0008-5472.CAN-10-1601

[bib47] Edgar R. Gene expression omnibus: NCBI gene expression and hybridization array data repository. Nucleic Acids Res 2002; 30: 207–210. 11752295 10.1093/nar/30.1.207 PMC99122

[bib48] Anders S, Huber W. Differential expression analysis for sequence count data. Genome Biol 2010; 11: R106. 20979621 10.1186/gb-2010-11-10-r106 PMC3218662

