# Supplementary material for: KDM2B, an H3K36-specific demethylase, regulates apoptotic response of GBM cells to TRAIL
Source: Cell Death Dis. 2017 Jun 29;8(6):e2897–. doi: 10.1038/cddis.2017.288 (PMC5520939; doi:10.1038/cddis.2017.288)
Supplement: Supplementary Information [file cddis2017288x1.docx]

**SUPPLEMENTARY INFORMATION**

**SUPPLEMENTARY FIGURES**

**Supplementary Figure 1.** qRT-PCR analysis of mRNA levels of other hits RING1A, NRF2, SUV39H2 and G9A in transduced cells. Expression levels were normalized to shControl cells.

**
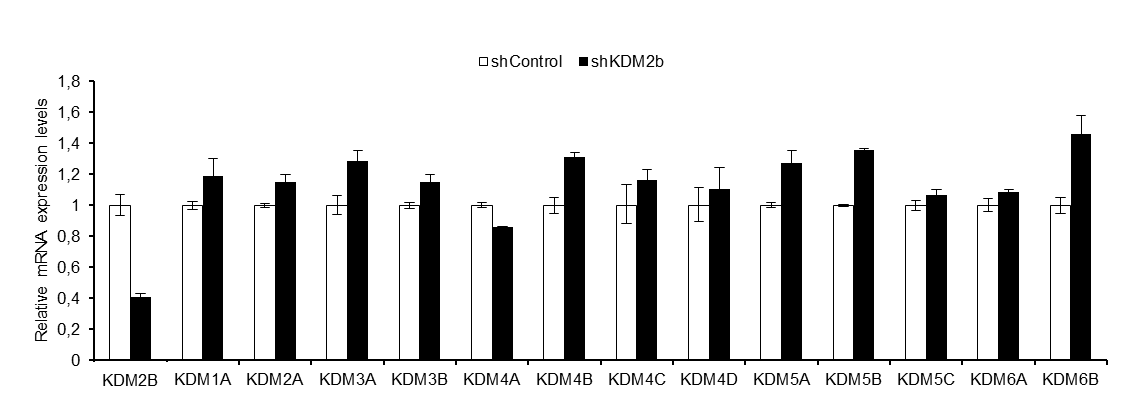
**

**Supplementary Figure 2.** qRT-PCR analysis of mRNA levels of KDM family members in KDM2B cells. Expression levels were normalized to shControl cells.


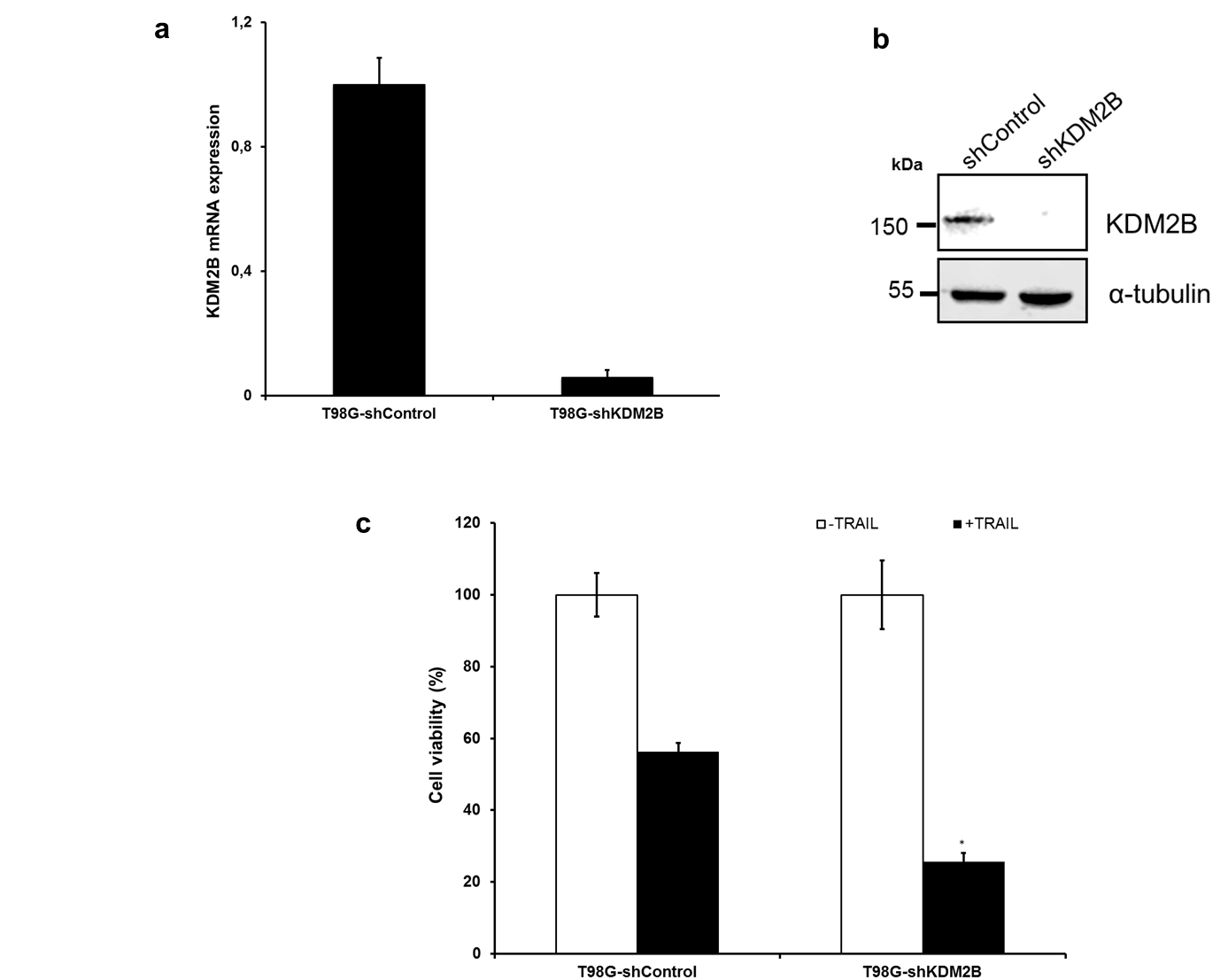


**Supplementary Figure 3.** (**a**) qRT-PCR analysis of KDM2B levels in T98G cells transduced with shControl or shKDM2B. Expression levels were normalized to shControl cells. (**b**) Western blot analysis of KDM2B protein levels in T98G cells transduced with shControl or shKDM2B. (**c**) Viability analyses of shControl or shKDM2B T98G cells upon TRAIL (50 ng/ml) treatment.


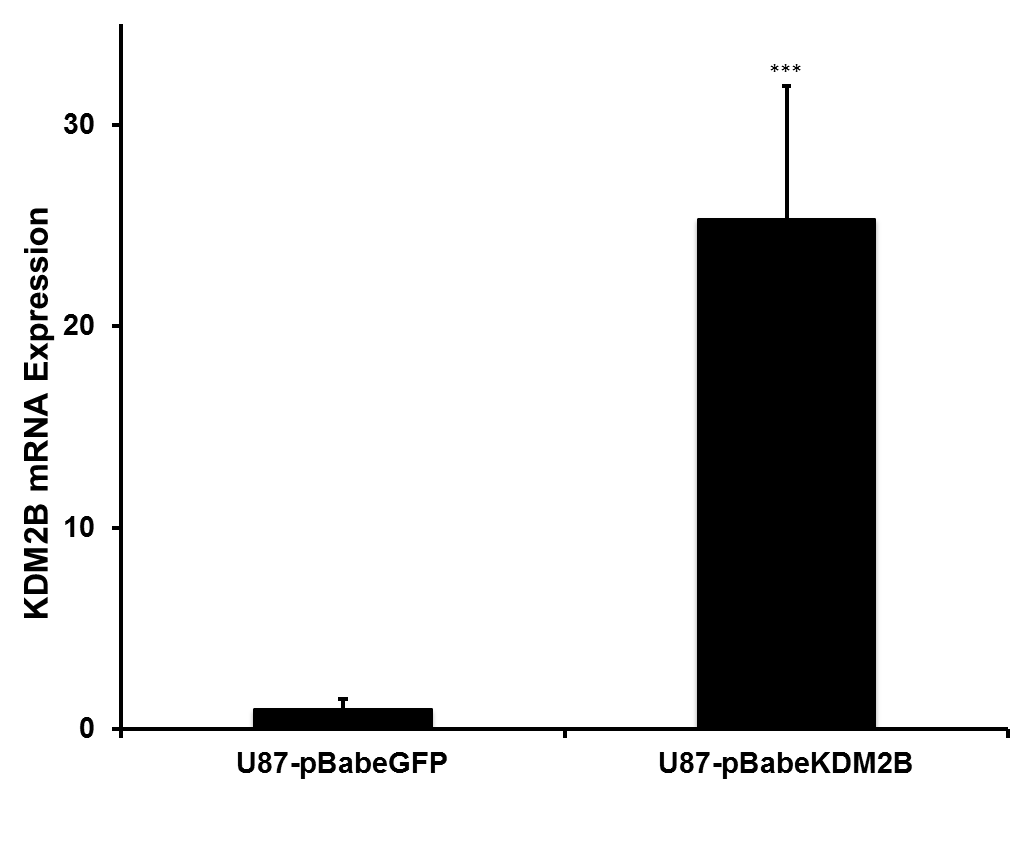


**Supplementary Figure 4.** qRT-PCR analysis of KDM2B mRNA levels in U87MG cells transduced with pBabeGFP or pBabeKDM2B. Expression levels were normalized to pBabeGFP cells.


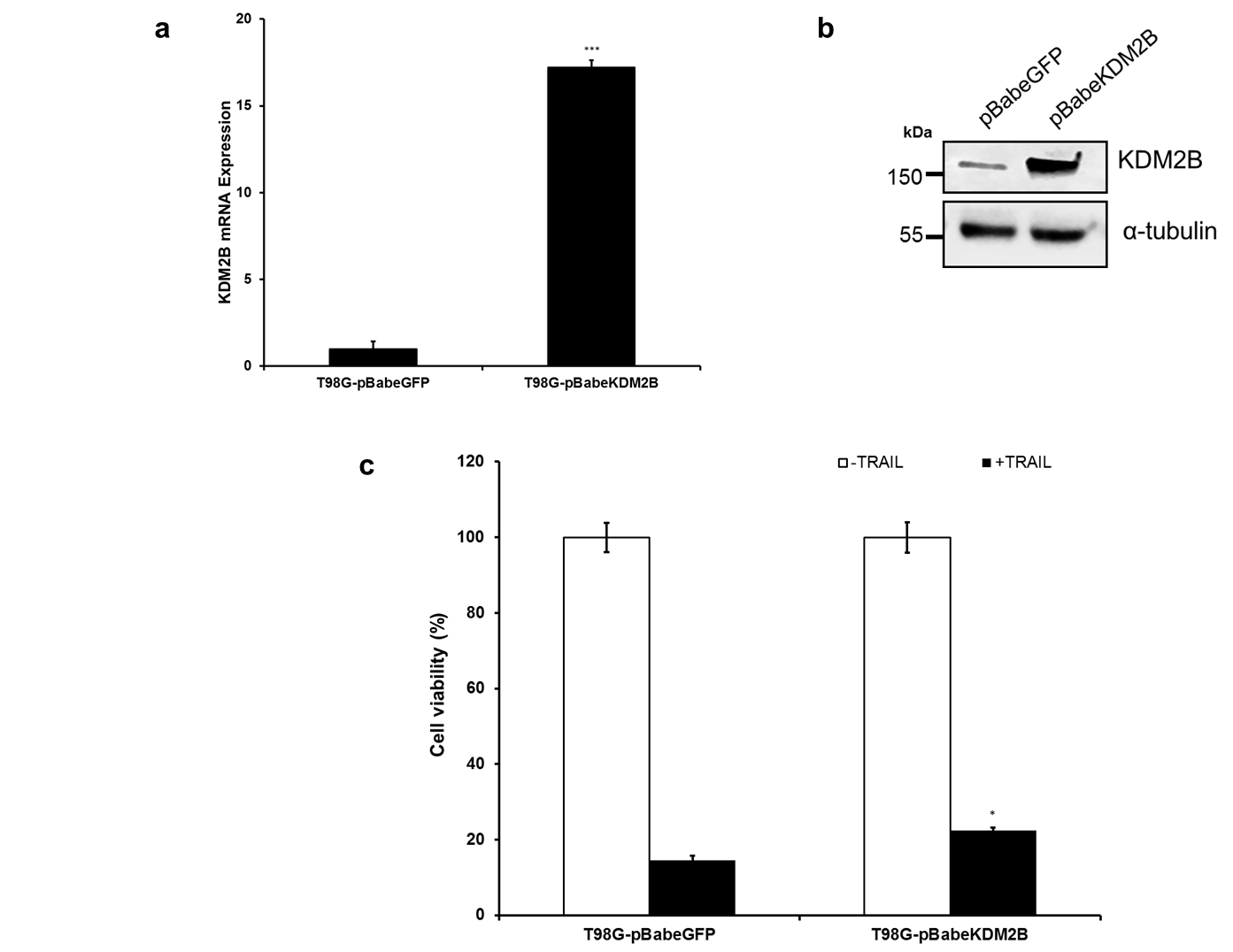


**Supplementary Figure 5.** (**a**) qRT-PCR and (**b**) western blot analysis of KDM2B levels in T98G cells transduced with pBabe-GFP or pBabe-KDM2B. Expression levels were normalized to shControl cells for qRT-PCR analysis. (**c)** Viability analyses of T98G cells transduced with pBabe-GFP or pBabe-KDM2B upon TRAIL (50 ng/ml) treatment.

**
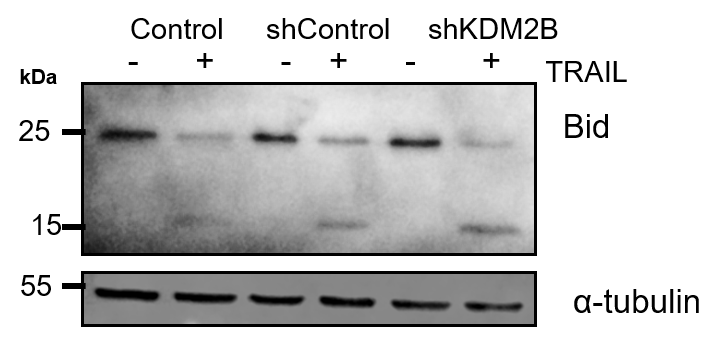
**

**Supplementary Figure 6.** Western blot analysis of total and cleaved Bid in control, shControl and shKDM2B cells treated or untreated with TRAIL.


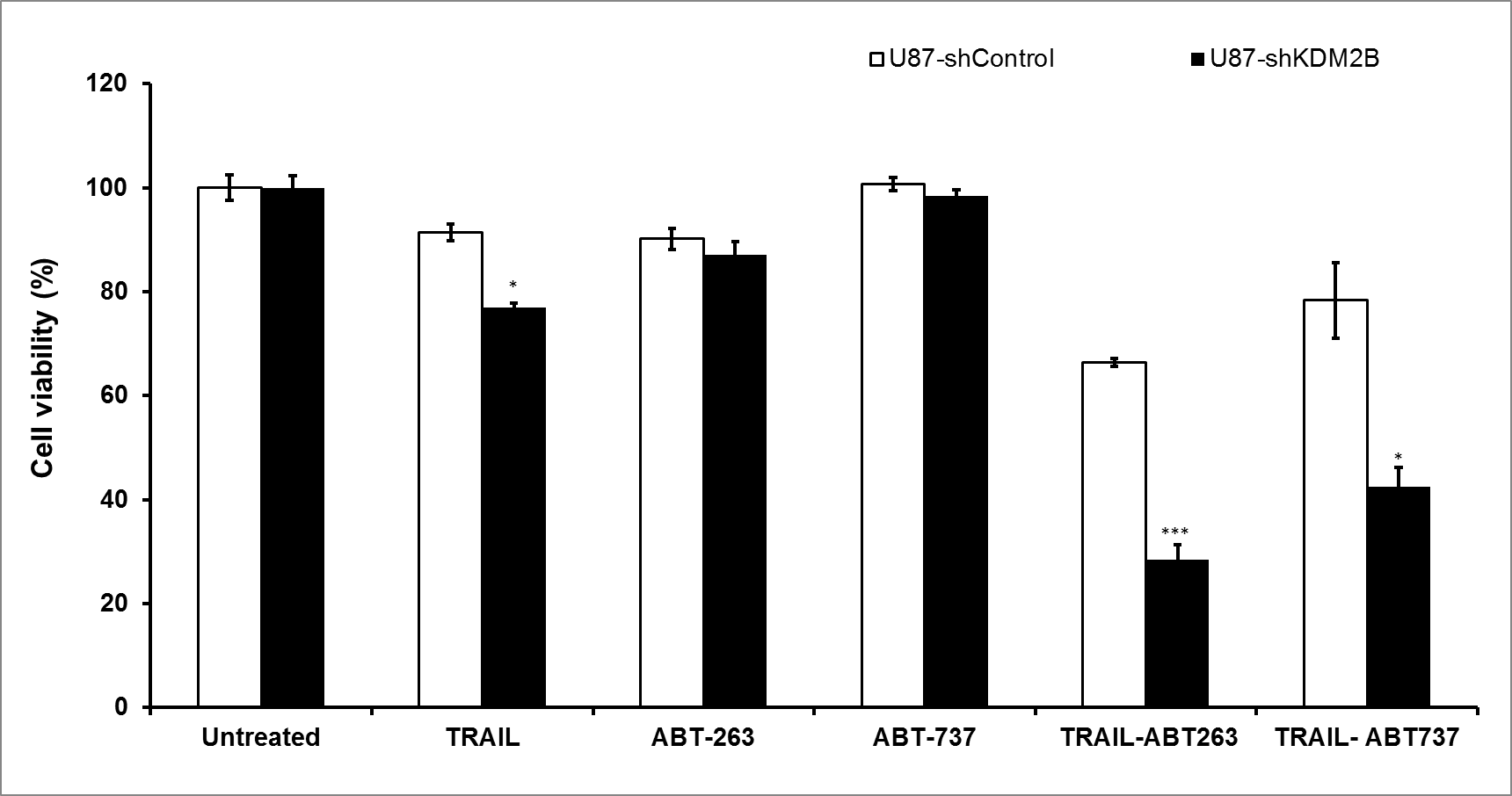


**Supplementary Figure 7.** Viability analysis of U87MG cells transduced with shControl or shKDM2B and treated with the indicated concentrations of TRAIL (50 ng/ml), ABT-263 (2.5 uM), ABT-737 (2.5 uM) and their combinations.

**
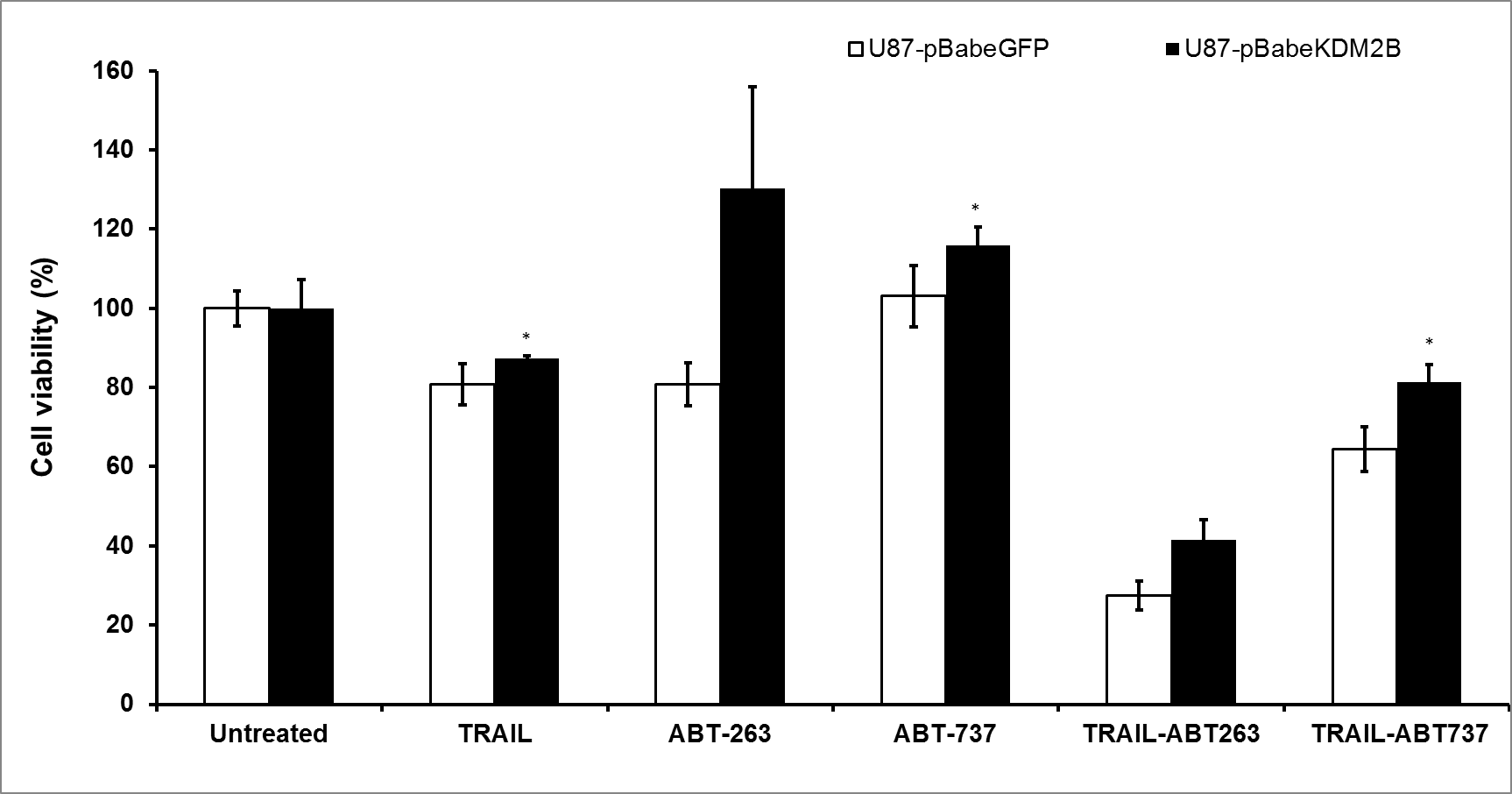
**

**Supplementary Figure 8.** Viability analysis of U87MG cells overexpressing GFP or KDM2B upon treatment with the indicated concentrations of TRAIL (50 ng/ml), ABT-263 (2.5 uM), ABT-737 (2.5 uM) and their combinations.


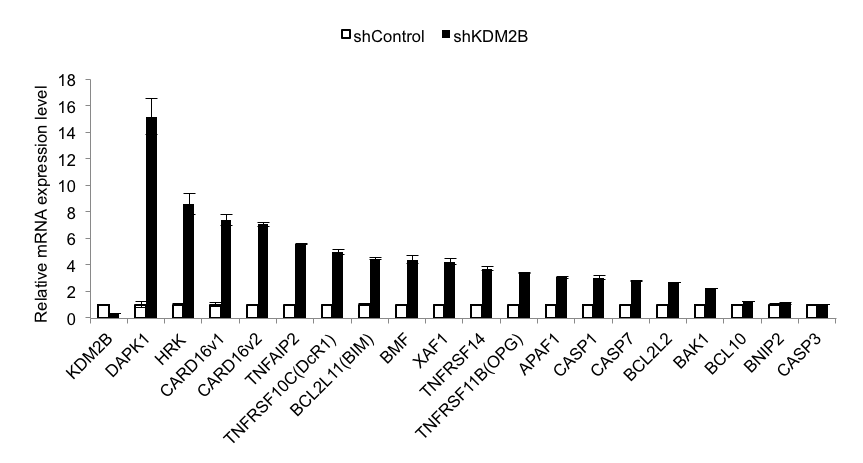

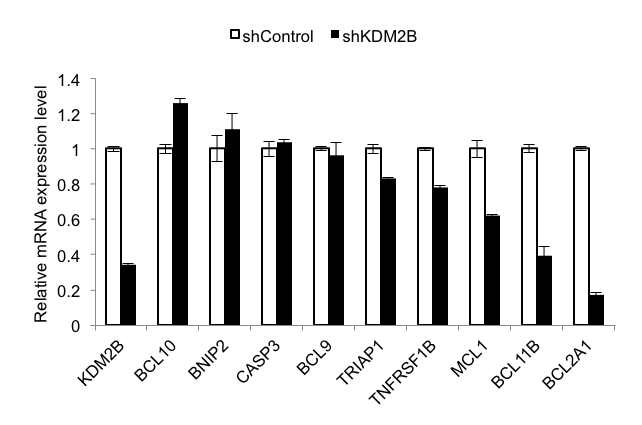


**Supplementary Figure 9.** qRT-PCR analysis of mRNA levels of top RNAseq results of shKDM2B cells normalized to shControl cells.


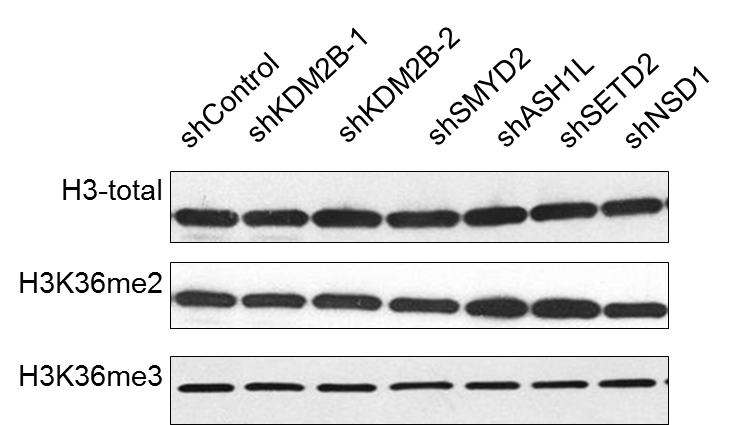


Supplementary Figure 10. H3K36me2 or H3K36me3 levels assessed by Western Blotting of histone extracts collected from shKDM2B, shSMYD2, shASH1L, shSETD2 and shNSD1 cells.

**
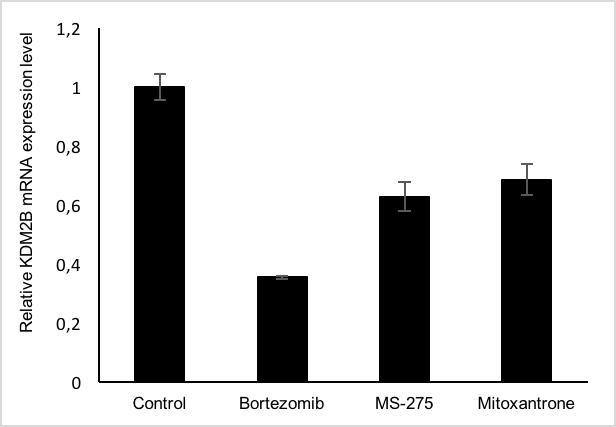
**

**Supplementary Figure 11.** q-RT-PCR analysis of mRNA levels of KDM2B in GBM cells treated with TRAIL-sensitizing agents, Bortezomib, MS-275 and Mitoxantrone. Expression levels were normalized to DMSO treated cells.

**Supplementary Table 1.** List of q-RT-PCR primers

**Supplementary Table 2.** Select sequences of shRNAs from the shRNA screen

**shKDM2B-1:** TGCTGTTGACAGTGAGCGCCCTGAACCACTGCAAGTCTATTAGTGAAGCCACAGATGTAATAGACTTGCAGTGGTTCAGGTTGCCTACTGCCTCGGA,

**shKDM2B-2:** TGCTGTTGACAGTGAGCGCTCAACCTGTCTGACTGCAATATAGTGAAGCCACAGATGTATATTGCAGTCAGACAGGTTGATTGCCTACTGCCTCGGA,

**shRING1A-1:** TGCTGTTGACAGTGAGCGACCCTGCAACGTCCCATCTATATAGTGAAGCCACAGATGTATATAGATGGGACGTTGCAGGGCTGCCTACTGCCTCGGA,

**shRING1A-2:** TGCTGTTGACAGTGAGCGACCAGCCAATAAGAGGACACAATAGTGAAGCCACAGATGTATTGTGTCCTCTTATTGGCTGGCTGCCTACTGCCTCGGA,

**shSMYD:** TGCTGTTGACAGTGAGCGACGGAGGGCCAAGCACTATAAATAGTGAAGCCACAGATGTATTTATAGTGCTTGGCCCTCCGGTGCCTACTGCCTCGGA,

**shNSD1:** TGCTGTTGACAGTGAGCGACCAGCTCGTCTCCTGCAAGAATAGTGAAGCCACAGATGTATTCTTGCAGGAGACGAGCTGGCTGCCTACTGCCTCGGA,

**shSETD2:** TGCTGTTGACAGTGAGCGCGCCATGTTACTTTGATCTTATTAGTGAAGCCACAGATGTAATAAGATCAAAGTAACATGGCATGCCTACTGCCTCGGA,

**shASH1L:** TGCTGTTGACAGTGAGCGCCCAAAGAACTTGGAGAACTATTAGTGAAGCCACAGATGTAATAGTTCTCCAAGTTCTTTGGATGCCTACTGCCTCGGA

**shHRK:**

TGCTGTTGACAGTGAGCGAAGCGATCGTAGAAACACAGAATAGTGAAGCCACAGATGTATTCTGTGTTTCTACGATCGCTCTGCCTACTGCCTCGGA

**SUPPLEMENTARY VIDEO LEGENDS**

**Supplementary Video 1.** U87MG GBM cells were transduced with shControl and treated with control medium at t=0. Images were acquired every minute for 23 hours on an Olympus Xcellence Pro inverse microscope.

**Supplementary Video 2.** U87MG GBM cells were transduced with shKDM2B and treated with control medium at t=0. Images were acquired every minute for 23 hours on an Olympus Xcellence Pro inverse microscope.

**Supplementary Video 3.** U87MG GBM cells were transduced with shControl and treated with TRAIL at t=0. Images were acquired every minute for 23 hours on an Olympus Xcellence Pro inverse microscope.

**Supplementary Video 4.** U87MG GBM cells were transduced with shKDM2B and treated with TRAIL at t=0. Images were acquired every minute for 23 hours on an Olympus Xcellence Pro inverse microscope.

**SUPPLEMENTARY MATERIALS & METHODS**

**q-RT-PCR analysis:** NucleoSpin RNA isolation kit (Macherey-Nagel,Germany) was utilized for extraction of RNAs from cultured cells as per of the manufacturer instructions. cDNAs were synthesized from 1 µg RNA using MMLV-RT enzyme (Invitrogen,USA), dNTP (Life Technologies, USA), hexanucleotide mix (Roche,Switzerland), and RNAsin (Promega,USA) as described ^1^. Quantitative RT-PCRs was carried out using the primers in **Supplementary Table 4** and GAPDH as housekeeping gene. SYBR Green (Roche,Switzerland)-based quantification was performed using Light Cycler 480 Instrument II (Roche,Switzerland).

**Relative KDM2B Gene expression levels of drug-treated U87MG:** TRAIL sensitizing agents Bortezomib (50 nM, Selleck Chemicals,USA), MS-275 (5 uM, Cayman Chemicals, USA) and Mitoxantrone (500 nM, Prestwick Chemicals, France) was added to U87MG cells and RNA was collected after 12 hours as described above.

**Cell viability assay with ABT-263 and ABT-767:** Cell viability was measured using ATP-based assays with CellTiter Glo (CTG) (Promega, Madison, WI, USA). The stable cell lines transduced with shControl, shKDM2B, pBabeGFP or pBabeKDM2B were seeded in 96 well plates as 10,000 cells/well. Next day, cells were treated with TRAIL (50 ng/ml), ABT-263 (2.5 uM), ABT-767 (2.5 uM) for 24 hours. The cells are also treated with medium containing combination of TRAIL and ABT-263 or ABT-737 for the same period of time. After 24 h treatment, media in the wells were withdrawn and 40 ul of CTG:media mix (1:10) per well was added. Bioluminescence was measured by BioTek’s Synergy H1 plate reader.

**Cell lysate preparation, Histone extraction, Immunoblotting:** Following treatment with TRAIL, U87MG cells were lysed with NP40 buffer [1% NP-40, 150 mM NaCl, 1 mM EDTA, 50 mM Tris-HCl (pH 7.8), 1 mM NaF, 0.5 mM PMSF and 1X protease inhibitor cocktail (cOmplete Protease Inhibitor Cocktail Tablets, Roche)] as described previously.^1^ For histone extraction, cells were harvested and resuspended in Triton Extraction Buffer (TEB: PBS containing 0.5% Triton-X-100 (v/v), 2 mM phenylmethylsulfonyl fluoride (PMSF), 0.02% (w/v) NaN3) at a cell density of 10^7^ cells per ml and lysed on ice for 10 minutes. The lysate was centrifuged at 6,500 x g for 10 minutes at 4^o^C. The pellet was resolubilized in 0.2N HCl as 4x10^7^ nuclei per ml. The solution was incubated at 4^o^C overnight. Next day, 1/5 volume of 1M NaOH was added into the solution to neutralize the pH. The mixture was centrifuged at 6,500 x g for 10 minutes at 4^o^C and the supernatant was collected. Protein quantifications of the lysates and histone extracts were performed by Pierce BCA Protein Assay Kit (Life Technologies, USA).

For Western Blotting, 10 µg of whole-cell extract proteins or 1 µg of acid histone extract proteins from each lysate were resolved on 4-15% gradient Mini-PROTEAN® TGX™ Gel (Biorad, US) and immunoblotted with antibodies against Histone H3 (Cell Signaling, US), H3K36me2 (Abcam, US), H3K36me3 (Cell Signaling, US), JHDM1B (Millipore, US), alpha-tubulin (Sigma-Aldrich, US), cleaved-caspase-3 (Cell Signaling, US), total caspase-7 (Cell Signaling, US), cleaved-caspase 8 (Enzo Life Sciences, US), HRK (Abcam, US), Bid (Cell Signaling, US) and cleaved-PARP (Cell Signaling, US) and detected by chemiluminescence after incubation with HRP-conjugated secondary antibodies (Santa Cruz, US).

**Immunohistochemistry:** Samples were fixed by 4% paraformaldehyde for 24 hours followed by 20% and 30% (wt/vol) sucrose treatment for cryosectioning. Consecutive cryosections (10 μm) were used for hematoxylin/eosin staining and fluorescent stainings. Vascular endothelial growth factor (VEGF) followed by fluorescent conjugated secondary antibody of Alexa fluor 488 GAM (Cell Signaling, US) was used for evaluation of vascular structures. Alexa fluor 488 conjugated annexin V (Dead Cell Apoptosis Kit, Life Technologies, US) was used for assessment of apoptotic activity. Dilutions were used as 1:50 for primary antibodies (Abcam, US) and 1:100 for the secondary antibody. All antibodies were incubated at 37°C for 2 hours. Hoechst (1μg/ml) was used in mounting medium. Images were taken under a Nikon Eclipse 90i confocal microscope and a Zeiss axioscope.

**RNA-sequencing and analysis:** Total RNAs of shControl and shKDM2B cells were isolated using NucleoSpin RNA isolation kit (Macherey-Nagel, Germany). Quality control was performed by Bioanalyzer for 2 biological replicates. The RNA integrity Number (RIN) of the samples was 10 for all samples. Construction and sequencing of RNA-seq library for each sample was carried out using TruSeq RNA-seq (polyA) Library Prep v2 based on protocols on Illumina HiSeq 2500 to generate 50 bp single-end reads. RNA sequence data was generated from the sequencer and converted to FASTQ files using the Illumina CASAVA 1.8.2 pipeline at the Weill Cornell Medical College (WCMC) Epigenomics Core. The reads that passed the quality control were aligned to human genome assembly hg19 using TopHat2^2^, which uses ultra-high-throughput short read aligner Bowtie as its core alignment engine and then analyzes the results to identify splice junctions between exons. The reads were annotated and counted for each transcript using Ensembl annotations available at the UCSC Genome Browser with R package GenomicFeatures^3^. The versions of the software used were TopHat2 v2.0.11, Bowtie v2-2.2.1 and Samtools v0.1.19.0. Around 18 million reads were acquired from sequencing of each sample that yielded in around 800 megabases. Around 83% of those reads passed filter quality check. Around 93% of those sequenced bases were equal or above Q score of 30.

**Statistical and functional analysis of RNA seq data and differentially expressed gene Identification**: Differentially expressed genes between shKDM2B and shControl were detected using DESeq^4^, which controls for Type I Errors (false-positives) by modeling the count data with extended Negative Binomial distributions, and thereby allows for general, data-driven relationships between variance and mean, and provides an effective algorithm for fitting the model to data. Note that each paired comparison, genes with mean read count in both sets of samples with fewer than 10 were filtered out; for the remaining genes, if the read count was 0 in either set (but not both of them), the read count was set to be 1 before input to R DESeq package for differential expression calculation. Significant differentially expressed genes for each pairwise comparison were selected with multiple-testing correction to maintain False Discovery Rate (FDR) at 5%. Genes with expression values that changed by a factor of 1.5 fold were deemed significant.

To relate the results to cell physiologic mechanisms, the complex biological processes induced by shKDM2B as compared to control were examined in the context of functional groups with Ingenuity Pathways Analysis (IPA), a web-delivered commercial application. Transcript identifiers were uploaded to IPA, each identifier was mapped to its corresponding gene object in the IPA Knowledgebase (www.ingenuity.com), which was searched for categories statistically enriched in the differentially expressed gene set, and the likelihood of perturbations in each category was scored.

**REFERENCES:**

1. Senbabaoglu, F. *et al.* Identification of Mitoxantrone as a TRAIL-sensitizing agent for Glioblastoma Multiforme. *Cancer Biol. Ther.* (2016). doi:10.1080/15384047.2016.1167292

2. Kim, D. *et al.* TopHat2: accurate alignment of transcriptomes in the presence of insertions, deletions and gene fusions. *Genome Biol.* **14,** R36 (2013).

3. Lawrence, M. *et al.* Software for Computing and Annotating Genomic Ranges. *PLoS Comput. Biol.* **9,** (2013).

4. Anders, S. & Huber, W. Differential expression analysis for sequence count data. *Genome Biol.* **11,** R106 (2010).
